# Machine Learning Aided Photonic Diagnostic System for Minimally Invasive Optically Guided Surgery in the Hepatoduodenal Area

**DOI:** 10.3390/diagnostics10110873

**Published:** 2020-10-27

**Authors:** Evgeny Zherebtsov, Marina Zajnulina, Ksenia Kandurova, Elena Potapova, Viktor Dremin, Andrian Mamoshin, Sergei Sokolovski, Andrey Dunaev, Edik U. Rafailov

**Affiliations:** 1 Research and Development Center of Biomedical Photonics, Orel State University, 302026 Orel, Russia; kandkseniya@gmail.com (K.K.); e.potapova@oreluniver.ru (E.P.); v.dremin1@aston.ac.uk (V.D.); dr.mamoshin@mail.ru (A.M.); dunaev@bmecenter.ru (A.D.); 2 Faculty of Information Technology and Electrical Engineering, University of Oulu, Optoelectronics and Measurement Techniques Unit, 90570 Oulu, Finland; 3 Aston Institute of Photonic Technologies, Aston University, Birmingham B4 7ET, UK; MZArea@hotmail.com (M.Z.); s.sokolovsky@aston.ac.uk (S.S.); e.rafailov@aston.ac.uk (E.U.R.); 4 Department of X-ray Surgical Methods of Diagnosis and Treatment, Orel Regional Clinical Hospital, 302028 Orel, Russia

**Keywords:** liver cancer, endogenous fluorescence, laser Doppler flowmetry, blood perfusion, minimally invasive interventions, machine learning

## Abstract

Abdominal cancer is a widely prevalent group of tumours with a high level of mortality if diagnosed at a late stage. Although the cancer death rates have in general declined over the past few decades, the mortality from tumours in the hepatoduodenal area has significantly increased in recent years. The broader use of minimal access surgery (MAS) for diagnostics and treatment can significantly improve the survival rate and quality of life of patients after surgery. This work aims to develop and characterise an appropriate technical implementation for tissue endogenous fluorescence (TEF) and assess the efficiency of machine learning methods for the real-time diagnosis of tumours in the hepatoduodenal area. In this paper, we present the results of the machine learning approach applied to the optically guided MAS. We have elaborated tissue fluorescence approach with a fibre-optic probe to record the TEF and blood perfusion parameters during MAS in patients with cancers in the hepatoduodenal area. The measurements from the laser Doppler flowmetry (LDF) channel were used as a sensor of the tissue vitality to reduce variability in TEF data. Also, we evaluated how the blood perfusion oscillations are changed in the tumour tissue. The evaluated amplitudes of the cardiac (0.6–1.6 Hz) and respiratory (0.2–0.6 Hz) oscillations was significantly higher in intact tissues (*p* < 0.001) compared to the cancerous ones, while the myogenic (0.2–0.06 Hz) oscillation did not demonstrate any statistically significant difference. Our results demonstrate that a fibre-optic TEF probe accompanied with ML algorithms such as k-Nearest Neighbours or AdaBoost is highly promising for the real-time in situ differentiation between cancerous and healthy tissues by detecting the information about the tissue type that is encoded in the fluorescence spectrum. Also, we show that the detection can be supplemented and enhanced by parallel collection and classification of blood perfusion oscillations.

## 1. Introduction

Abdominal cancer is a widely prevalent group of tumours with a high level of mortality if diagnosed at a late stage. Although the cancer death rates have in general declined over the past few decades, the mortality from tumours in the hepatoduodenal area specifically has increased in recent years [1]. Moving towards the broader use of minimally invasive techniques for diagnosis and treatment is promising to significantly improve the survival rate and quality of life of patients after surgery. Minimally invasive techniques require the use of reliable tools for real-time feedback to assist the surgeon. Usually, the manipulations in the operation field are conducted under ultrasound or X-ray control and visualisation. While the methods can provide an exceptional accuracy of the tool positioning, they hardly give sufficient information on the tissue type and, specifically, on the tumour borders. Various approaches have been put forward to solve this issue. The optical methods have been widely investigated in that respect and demonstrated high diagnostic potential in many applications [2,3,4,5]. Despite the gradual transition from the in vivo single-point measurements towards the in vivo imaging techniques, the use of fibre optical probes for the surgical guidance has great potential to compete with the costly techniques and to significantly improve the outcome of the routine procedures for cancer eradication. Most imaging approaches still need to address major issues concerning the motion artefacts, real time imaging processing and the trade-off between the parameters of resolution, frame rate and aperture diameter [6]. In particular, this set of limitations affects the minimally invasive surgical interventions as one deals with the tools of low outer diameters being applied in confined space of the abdominal cavity [7].

In recent years, there has been considerable interest in the measurements of the fluorescence parameters to be applied for the diagnostics in minimally invasive surgery [8]. The methods are based on the recording of the endogenous fluorescence. However, they often require the intravenous injection of fluorescence dyes or molecular fluorescence probes specifically binding to tumour cells [9,10]. The association between TEF emission and changes in tissue metabolism as well as the blood circulation and histological architecture offers a powerful diagnostic tool for direct monitoring of the malignancy of studied biotissues. TEF, based on autofluorescence spectra registration, can provide comprehensive information about the physiological or altered morphofunctional properties of cells and tissues, including ones caused by oncology [11]. Due to high proliferation, tumour cells have increased metabolic needs compared to normal ones [12], and tumour metabolism is associated with changes in relative concentrations of NADH and FAD [13]. These endogenous fluorophores of cells and tissues can serve as biomarkers for studying differences between tumour and non-tumour areas [14,15,16,17,18].

In the traditional approach, the TEF measurements were used to evaluate the changes in tissues based on the comparison of the absolute values of the related emission parameters in tumour and non-tumour areas. Nowadays, research tends to focus on machine learning (ML) approaches that combine input feature sets in one or several diagnostic classifiers [19]. One promising application of ML is MAS as the medical procedure commonly requires a high speed of the onsite decision making for the surgeon. More recent evidence shows that the diagnostic techniques based on the measurements of the intrinsic fluorescence aided with the machine learning algorithms can be used for distinguishing malignant and benign colorectal tissue [20].

To the best of our knowledge, the applicability of ML in fluorescence liver cancer diagnostics has not yet been studied thoroughly. The available published results in this research area either focus on the MRI or CT studies [21], are limited using tumour-targeting fluorescent labels [22] or reported results obtained ex vivo [23].

Recently, authors have published data on the evaluation of the liver tumours with the needle optical probe combining TEF and diffuse reflectance measurements [24]. Except for that, despite high interest and potential outcome, no one, to the best of our knowledge, has demonstrated results of the ML application for cancer diagnosis that deploys data obtained by optical probe in vivo and in situ in the hepatoduodenal area.

In this work, we present results of the ML techniques to facilitate optically guided surgery on tumours by the detection and classification of cancerous and healthy tissues. The data that we use in our studies was obtained in vivo and *in situ*. In particular, we focus on cancers of the liver and bile duct and collect and classify the data for TEF and blood perfusion. The focus on two types of data has the following reason: it is known that in vivo recorded fluorescence spectra are influenced by many factors such as the absorption by blood in the living tissue [25]. This blood-induced absorption attenuates and changes the shape of the fluorescence signal [26]. To mitigate this problem, we additionally analyse the parameters of the blood perfusion measured in living intact tissue (cf. [27]). For this, we deploy laser Doppler flowmetry (LDF). LDF provides a powerful tool for the blood perfusion measurements and analysis of rhythmic oscillations in the fine structure of microvascular blood flow. It was demonstrated that LDF is a highly informative method across a range of medical conditions and general physiological health monitoring applications. To record TEF and LDF blood perfusion parameters during MAS in patients with cancers of liver and bile duct, we implemented a flexible optical fibre probe capable of measurements in the hepatoduodenal area. We used ML techniques to detect the tumour borders by classification of healthy and cancerous tissues using the TEF and LDF data. The general aim of this work was to develop an appropriate technical implementation for TEF and LDF measurements and to assess the efficiency of ML methods for real-time diagnosis of liver cancer and tumours in the hepatoduodenal area.

## 2. Materials and Methods

### 2.1. Setup and In Situ Data Collection

**Figure 1:**
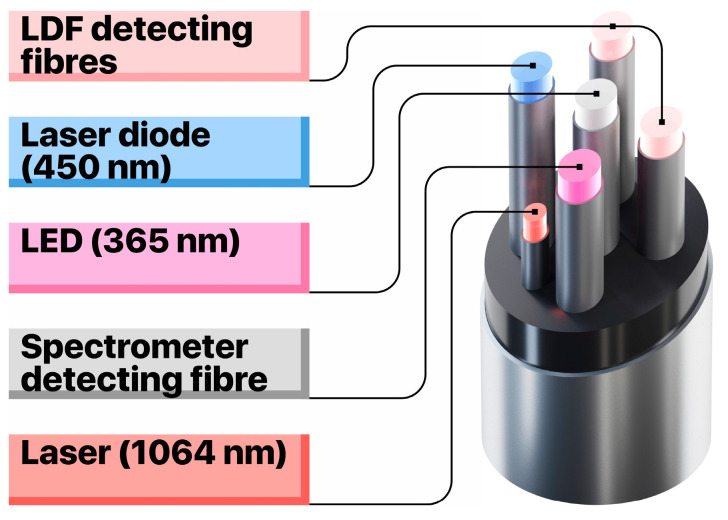
The configuration and measuring channels of the used fibre-optic probe.

**Figure 2:**
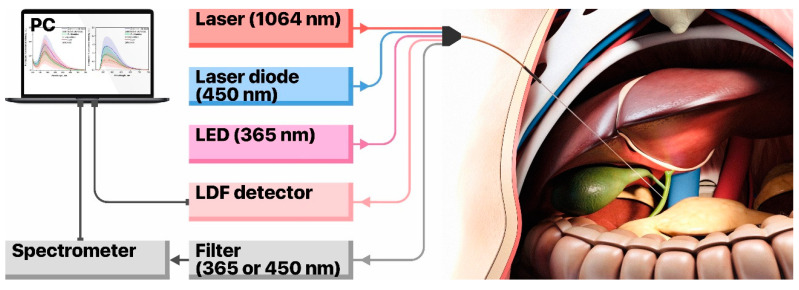
The components of measuring setup and probe placement in the region of interest.

The setup to collect data on TEF and LDF consists of a custom-built, flexible fibre-optical probe, two LED sources for fluorescence excitation (at 365 nm and 450 nm), a set of fluorescent filters, a CCD-based spectrometer and a laser Doppler flowmetry channel for blood perfusion measurements that utilises a single-mode laser at 1064 nm. The prototype of the fibre-optical probe (Figure 1) has two emitting fibres to excite TEF at wavelengths of 365 nm and 450 nm and a fibre to collect the fluorescence emission (all fibres are of 400 µm in diameter). Moreover, the probe is equipped with one emitting single-mode optical fibre and two multimode collecting fibres for the LDF channel. This configuration enables to register the fluorescence of all major endogenous fluorophores in living tissue, as well as to record the level of blood perfusion in the area of interest (Figure 2).

**Figure 3:**
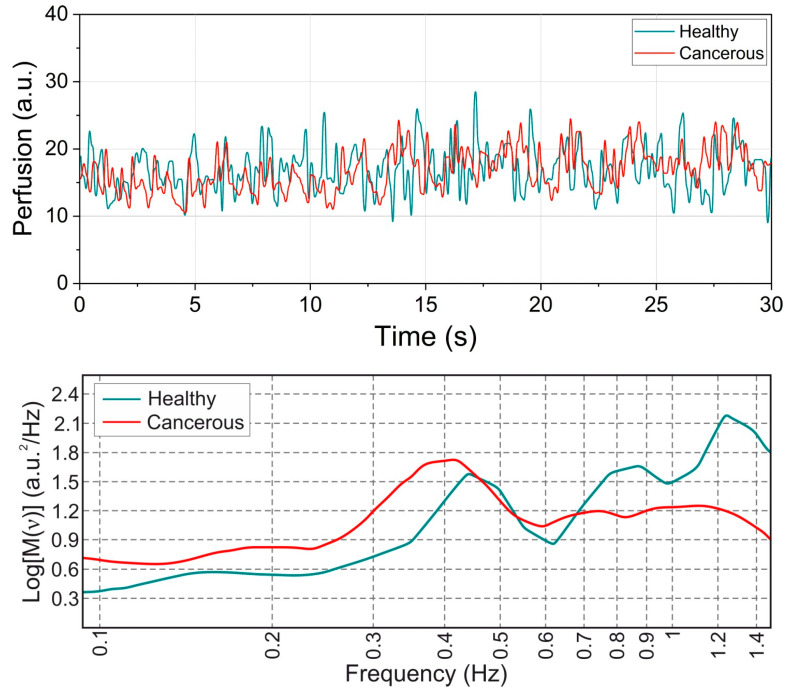
Exemplary blood perfusion records (upper panel) from the intact bile duct wall and the tumour located in the bile duct and the corresponding integrated wavelet spectra (IWS; bottom panel).

The proposed setup was validated in the framework of limited tests in patients with obstructive jaundice caused by liver cancer. The patient cohort comprised of 27 volunteers. The measurements from the LDF channel were used as a sensor of the tissue vitality. The TEF spectra were recorded primarily in the areas of interest demonstrated a stable level of blood perfusion of about 15 a.u. Figure 3 (top panel) shows the exemplary records of blood perfusion registered in the intact bile duct and in the cancer block. This procedure allowed us to avoid areas of necrosis and to decrease the TEF data variation associated with the variable blood volume fraction in the tissue. The measurements were performed during planned diagnostic and therapeutic interventions under ultrasound and X-ray examination. For every patient, 8–10 spectra were taken at the excitation wavelength of 365 nm and 450 nm. They were equally split between cancerous and intact tissues. In that way, 4–5 spectra were measured for each class in a patient. The measurement points were evenly distributed over the area of interest except for places with low blood perfusion prominent necrosis or covered with blood. Also, two 1 min long LDF recordings were made for places with apparent tumour and normal tissues respectively. The studies were conducted at the department of interventional radiology of Orel Regional Clinical Hospital (Orel, Russia) and were approved by the local Committee for Human Biomedical Research Ethics (record of the meeting No. 10 of 16.11.2017).

### 2.2. Data Collection

The main challenge of the proposed technique lies in the collection of TEF data under the X-ray control directly during a surgical procedure. The imaging procedure, being realised with a radiopaque substance that fills the cavity of the bile duct, allows the surgeon to visualise the contour of the walls and the tumour block. In this case, the 2D radiological control is the only piece of feedback information about the probe position with respect to the bile duct and the tumour itself. Actually, this indirect approach might be sufficient for MAS as such. However, from the point of view of the data collection quality for ML, the indirect control might be insufficient for a precise localisation of the sampling point and, as a result, for a right labelling of the TEF spectrum. Thus, a spectrum coming from a healthy tissue can be falsely labelled as cancerous and vice versa. When used for training and testing of ML models, such spectra contribute towards a high value of the intrinsic lowest possible error (LPE) in classification and make it difficult to evaluate the performance of the ML models and to interpret their prediction results.

Besides the difficulty of the probe positioning due to the limitations of the available 2D X-ray visualisation (i), we identify three other sources of possible data mislabelling: (ii) the lack of ability to reliably visualise blood and bile in the field of view of the fibre-optical probe which significantly affects the quality of the collected fluorescence spectra; (iii) the uncertainty of the distribution of the cancer cells over the mucous surface; and (iv) the effects of the probe pressure on the tissues that manifests itself in the variable blood volume fraction in the tissue.

In the presented study, all patients were diagnosed with cancer and their samples were verified morphologically by biopsy. Nevertheless, regarding the challenges (i) and (iii), it should be noted that the allocation of the areas with cancer tissue significantly varies between the patients. The X-ray visualisation helps to identify the tumour block and the position of the probe. At the same time, in the vicinity of the tumour block, the malignant cells can belong to the primary tumour originated from the epithelial tissues of the bile duct or cells proliferated from the surrounding organs. Also, in some cases, the cancer cells can be absent on the surface of the cancerous tissues. This happens when the tumour block compresses the healthy tissue where the probe is placed but does not permeate through the wall of the organ. These aspects further contribute to the increase of the system’s LPE by leading to false labelling of spectra used for the training and testing of ML models.

### 2.3. Machine Learning Method

**Figure 4:**
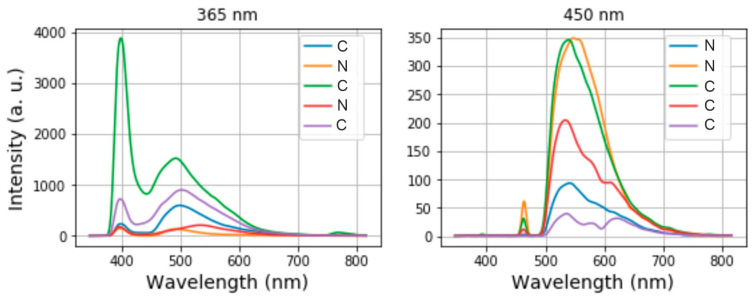
5 arbitrary fluorescence spectra and their labels obtained at the excitation channel of 365 nm (**left**) and 450 nm (**right**). In these graphs, the label “N” denotes a normal tissue whereas “C” a cancerous tissue.

In our experiments, the spectrometer for the TEF measurements covers the range between 346 nm and 816 nm sampled with 2100 pixels. To build and determine suitable machine learning models for classification of healthy and cancerous tissues, we consider the intensity spectra taken at 365 nm and 450 nm separately. For the purposes of classification, the following labels were used to label the spectra: “C” for cancerous tissues and ”N” for healthy tissues. Figure 4 shows 5 arbitrary spectra and their labels for each channel. On the left side of each spectrum, we see a sharp excitation peak and to its right, a broad fluorescence spectrum. The information on whether a tissue is healthy or cancerous is encoded in the intensity of the fluorescence peak, its position in the spectrum and its shape. We use a Python-based library called Scikit-learn to pre-process and to classify the spectra automatically by ML methods.

In terms of data pre-processing, we first removed the data samples where the noise floor overlapped with the actual fluorescence spectra. To further reduce the influence of noise, the remaining spectra were smoothed using a FIR filter. In the next step, noisy, not informative sections at the beginning (blue sides) of the spectra as well as at their (red) ends were cut off. This resulted in the reduced dimensionality of each spectrum of (1, 1920) instead of the initial dimension of (1, 2100) that was determined by the spectrometer pixel number.

The training and test sets were randomly split as follows: 199 samples for training and 40 samples for testing in the case of the 356 nm channel and 120 samples for training and 40 samples for testing in the case of the 450 nm channel. To increase the performance of our ML models, the data was standardised by subtracting the data mean value and dividing the result by the standard deviation.

**Figure 5:**
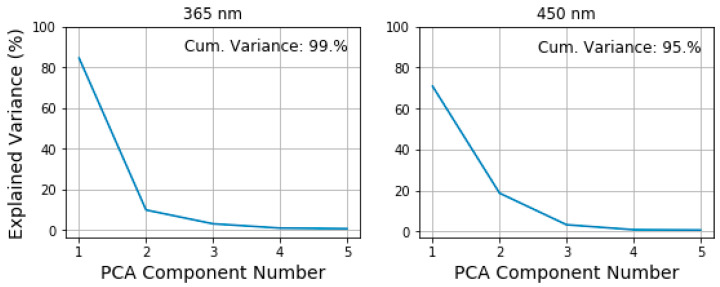
Example of percentage of explained variance at the excitation channel of 365 nm (**left**) and 450 nm (**right**) achieved by PCA.

To further reduce the dimensionality of our training and test data, we applied the principal component analysis (PCA) with 5 principal components. As Figure 5 shows, 5 principal components retain more than 90% of the information stored in the spectra. The first two components having the highest values carry the biggest part of this information. We interpret the first two components as the indicators of the fluorescence peak intensity and its position in the spectrum. After the PCA is performed, the dimension of each spectrum is only (1, 5). In our statistical experiments, we observe a variation of the first two PCA components values by ±3% and much smaller variation in other components. However, this variability does not prevent us from seeing a clear difference between the first and the second component.

Five classification models and their performance in differentiating between the healthy and cancerous tissues were considered for the TEF spectra of each channel as well as for the LDF blood flow oscillations. Those are the k-Nearest-Neighbours (KNN) algorithm [28], a Decision Tree (DT) [29], Support Vector Machines (SVM) [30] and such ensemble methods as the Random Forest (RF) [31] and AdaBoost [32]. In the blood perfusion analysis, the logistic regression model was applied instead of AdaBoost. A 10-fold validation was applied to evaluate the accuracy of each method. The accuracy denotes the ratio between the rightly classified values, and all considered ones and measures how close the predicted value of the class is to the true class label. Two further metrics that we used to evaluate the classifiers’ performance are the sensitivity and the specificity. The sensitivity is the ratio between the true positives and the sum of true positives and false negatives, i.e., the portion of actual positives that are correctly predicted. The specificity measures the number of true negatives put into relation to the sum of true negatives and false positives, it is the portion of actual negatives that are correctly predicted [33]. The sensitivity is an important measure to accurately diagnose the cancerous tissues, whereas the specificity determines how well the system detects healthy tissues. In an ideal case, both sensitivity and specificity tend towards 100%. In the following, all three metrics (accuracy, sensitivity and specificity) are evaluated as an average of 10 statistical experiments for which the data was randomly shuffled each time.

When looking at Figure 3, we can assume that the information of the tissue type is encoded in the frequency components of the blood perfusion time series. The oscillations in the cancerous tissue are slower whereas the time series collected from healthy tissue exhibits more high-frequency components. To validate the assumption and extract this information, we pre-process the LDF data by means of the wavelet analysis with the Morlet wavelet taken as a mother function. We calculated the integrated wavelet spectra (IWS) using the signal analysis technique in the frequency domain. Examples of representative IWS for cancerous and normal tissues are presented in Figure 3 (bottom panel). The length of the recordings allowed us to calculate 3 spectral ranges of the oscillations in the fine structure of capillary blood flow corresponding to the cardiac (0.6–1.6 Hz), respiratory (0.2–0.6 Hz) and myogenic activities (0.2–0.06 Hz) [34]. The maximal amplitude of the oscillations in every spectral range was taken as a diagnostic parameter. One-Way ANOVA with Tukey’s post hoc test was used to check the significance of the statistical variation between blood perfusion parameters measured in the tumour and the intact tissue. Only demonstrated statistically significant difference parameters of blood perfusion oscillations were used as the feature space for the ML methods.

## 3. Results

As in the case with data pre-processing, the data evaluation for TEF was done using the Scikit-learn library. The considered ML models underwent initial tuning in terms of the best prediction accuracy to pick the best possible hyperparameters. The resulting KNN classifier had 4 neighbours with distance weighting for both channels; also for both channels, the best maximum depth of the decision tree turned out to be only 1; the SVM model had a regularisation parameter of *Z* = 0.1 and a linear kernel for both 365 nm and 450 nm; the RF classifier for the 365 nm channel consisted of 5 estimators with a depth of 3; whereas the RF model for the 450 nm channel had 7 estimators with a depth of 3; the AdaBoost model consisted of 5 sub-estimators for each channel.

Table 1 and Table 2 summarise the results for accuracy, sensitivity and specificity we achieved for 10 statistical experiments where the data used for training and testing was randomly shuffled each time. The results are presented as a mean value ± standard deviation in percent.

As Table 1 shows, the results of the TEF classification exhibit higher values for the 450 nm channel than for the 365 nm channel for all considered metrics. This suggests dropping the 365 nm channel in future which might considerably simplify the setup. Additionally, the dropping of the 365 nm channel would increase the in-field practicability of the suggested method. This is because the excitation radiation of 365 nm lying within the UV spectrum induces higher phototoxicity for living tissues than the light at 450 nm [35]. For TEF, the accuracy varies between 60% and 67% for the 365 nm channel and between 62% and 72% for the 450 nm channel.

As discussed in Section 2.2, we assume a high LPE implicating that several spectra were falsely labelled right at the beginning. We assume an LPE of around 15%. In this case, the accuracy is limited by this value, meaning that even a perfect classifier would not be able to achieve better accuracy results than around 85%. From this point of view, to achieve accuracy values up to 72% is highly promising.

In detail, from all considered values of accuracy, the SVM algorithm for the TEF data provides the best performance. Moreover, in terms of specificity, SVM shows the best results, going to 96–99% well predicting the negative cases, i.e., the cases of healthy tissues. The second-best result is shown by the DT model. However, if you consider the sensitivity values, both SVM and DT perform poorly on the prediction of cancerous tissues, which is the most important aspect for in situ diagnosis. Taking into account the value of sensitivity, the best performance is shown by KNN for both channels and AdaBoost, specifically for the 450 nm channel. The considered Random Forest performs poorly in terms of all three metrics.

In general, we observe a high variety in sensitivity values that manifests itself in high values of standard deviation. This occurs due to the difficulties of the data collection described in Section 2.2 and poses the requirement to improve the data collection and labelling in further stages of the approach development.

**Figure 6:**
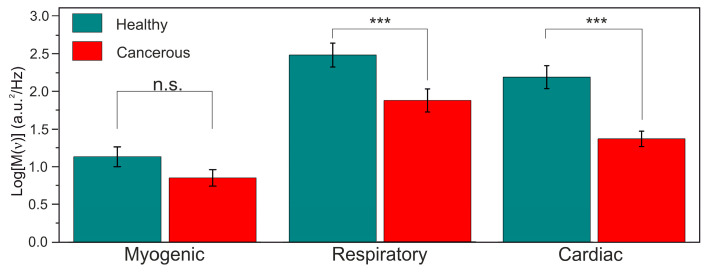
Results on the statistical analysis (One-Way ANOVA with Tukey’s post hoc test, *** *p* < 0.001, n.s.: not significant, Mean ± SE) of the differences in the oscillations in normal intact and cancerous tissues in the hepatoduodenal area.

As for LDF, the evaluated amplitudes of the cardiac and respiratory oscillation were significantly higher in intact tissues (*p* < 0.001) compared to the cancerous ones, while the myogenic oscillation did not demonstrate any statistically significant difference. The results on the statistical analysis of the differences in the oscillations in normal intact and cancerous tissues are presented in Figure 6. The reason for the observed distinction of the amplitude values in the healthy and cancerous tissues can lie in the mechanical properties of the tumour resulting in the dimming of the amplitude of the pulse that propagates through the microvessels within the cancerous tissue.

The results obtained by the ML models applied to the values of blood perfusion oscillations obtained from wavelet transform (Table 2) suggest that the parameters especially of the cardiac oscillations are of high a diagnostic value and can effectively supplement the TEF analysis and classification. However, more clinical research and better feature extraction as well as the fine-tuning of the ML models are needed to produce better prediction results and to correctly interpret them.

As Table 2 presents, the best results are achieved by logistic regression applied to classify the cardiac oscillations. This suggests focussing only on the data obtained for cardiac oscillations and to use logistic regression to evaluate it in future. Further, reliable evaluation of the blood perfusion pulsations associated with the heart activity requires as much as twice shorter LDF recording duration than the one required for the respiratory oscillations. As a result, the duration of the LDF recording can be shortened.

## 4. Discussion

The challenge of more accurate data collection to significantly minimise the LPE still requires an appropriate solution. Further development of the proposed multimodal approach by equipping it with additional optical methods (e.g., diffuse reflectance spectroscopy or optical proximity sensor) can be an option to improve the accuracy of the probe placement. An approach based on the diffuse reflectance measurements might allow for a significant decrease in the influence of blood absorption on the recorded TEF spectra.

A non-contact proximity sensor embedded in the probe combined with the subsequent automation of the data collection at a fixed optimal distance might eliminate the negative effects of the sensor tip pressure on the soft tissues under study. Moreover, the saline delivery subsystem for the pus, mucus and blood removal from the tissue surface and optical probe cleaning during the procedure are also considered to be promising for the improvement of the quality of the data collection.

Based on the achieved results, we believe that the use of the 365 nm channel can be dropped in the further design development of the fibre-optic probe since it does not contribute to the overall performance. We see that TEF analysis and classification can be assisted by the analysis of the LDF data for cardiac oscillations. However, deeper-going studies with respect to the suitable feature extraction and ML model hyper-parameters are needed to effectively supplement the TEF classification with LDF classification. To improve the data quality and efficiency for the LDF analysis, the length of the blood perfusion recordings can be shortened from 1 min to 30 s as it is enough for the suggested analysis of the cardiac oscillations. As for the ML methods, AdaBoost and KNN models demonstrate the best results in terms of accuracy, specificity, and sensitivity and should be considered for classifying cancerous and noncancerous tissues in real-time in situ TEF diagnosis. The logistic regression is preferable for the classification based on the blood perfusion oscillations data.

## 5. Conclusions

Our results clearly demonstrate that a fibre-optic TEF probe accompanied with ML algorithms such as k-Nearest Neighbours or AdaBoost is highly promising for real-time in situ differentiation between cancerous and healthy tissues by detection the information about the tissue type that is encoded in the fluorescence spectrum. This detection can be supplemented and enhanced by parallel collection and classification of blood perfusion rhythmic data, especially the one that denote cardiac oscillations. Clearly, the data collection procedure as well as the design of the proposed fibre-optic probe have to be improved. Once it is done, we are convinced that the proposed fibre probe together with the elaborated ML techniques constitutes a highly promising device for a prompt and precise in situ decision-making and would allow to choose the optimal surgical tactics during the tumour resection.

As a next step, we will elaborate a procedure for the collection of data with much higher quality as well as improve the design of the fibre-optic probe by, for instance, dropping the 365 nm excitation channel and introducing a non-contact proximity sensor.

## Figures and Tables

**Table 1:** Achieved results of Accuracy and Specificity in % for each classifier and each excitation channel for TEF measurements.

*Channel*	*365 nm*			*450 nm*		
Model	Accuracy	Sensitivity	Specificity	Accuracy	Sensitivity	Specificity
KNN	60 ± 8	41 ± 21	73 ± 12	62 ± 6	46 ± 21	78 ± 8
Decision Tree	67 ± 7	28 ± 25	89 ± 9	67 ± 8	27 ± 37	94 ± 13
SVM	67 ± 8	15 ± 28	96 ± 6	72 ± 6	28 ± 35	99 ± 2
Random Forest	65 ± 8	27 ± 27	86 ± 11	64 ± 7	28 ± 28	88 ± 8
AdaBoost	65 ± 6	26 ± 26	87 ± 7	64 ± 5	37 ± 37	86 ± 16

**Table 2:** Results of Accuracy and Specificity in % for each classifier for blood perfusion rhythmic oscillations.

*Channel*	*Cardiac Oscillations*			*Respiratory Oscillations*		
Model	Accuracy	Sensitivity	Specificity	Accuracy	Sensitivity	Specificity
KNN	58 ± 7	34 ± 26	82 ± 16	56 ± 10	35 ± 10	77 ± 18
Decision Tree	62 ± 7	61 ± 22	65 ± 24	55 ± 6	37 ± 20	72 ± 18
SVM	60 ± 9	26 ± 20	93 ± 8	59 ± 8	36 ± 19	81 ± 13
Random Forest	54 ± 15	57 ± 17	51 ± 30	58 ± 6	42 ± 14	73 ± 13
Logistic Regression	70 ± 7	52 ± 9	87 ± 9	64 ± 8	49 ± 11	79 ± 12
